# Unconventional Stoichiometries of Na–O Compounds at High Pressures

**DOI:** 10.3390/ma14247650

**Published:** 2021-12-12

**Authors:** Lihua Yang, Yukai Zhang, Yanli Chen, Xin Zhong, Dandan Wang, Jihui Lang, Xin Qu, Jinghai Yang

**Affiliations:** 1 Key Laboratory of Functional Materials Physics and Chemistry of the Ministry of Education, National Demonstration Center for Experimental Physics Education, College of Physics, Jilin Normal University, Siping 136000, China; yanglh@jlnu.edu.cn (L.Y.); Lance19950815@163.com (Y.Z.); zhongxin@calypso.cn (X.Z.); mila880227@126.com (D.W.); jhlang@jlnu.edu.cn (J.L.); 2 State Key Laboratory of Integrated Optoelectronics, College of Materials Science and Engineering, Jilin University, Changchun 130012, China

**Keywords:** high pressure, structure prediction, electride

## Abstract

It has been realized that the stoichiometries of compounds may change under high pressure, which is crucial in the discovery of novel materials. This work uses systematic structure exploration and first-principles calculations to consider the stability of different stoichiometries of Na–O compounds with respect to pressure and, thus, construct a high-pressure stability field and convex hull diagram. Four previously unknown stoichiometries (NaO_5_, NaO_4_, Na_4_O, and Na_3_O) are predicted to be thermodynamically stable. Four new phases (*P*2*/m* and *Cmc*2_1_ NaO_2_ and *Immm* and *C*2*/m* NaO_3_) of known stoichiometries are also found. The O-rich stoichiometries show the remarkable features of all the O atoms existing as quasimolecular O_2_ units and being metallic. Calculations of the O–O bond lengths and Bader charges are used to explore the electronic properties and chemical bonding of the O-rich compounds. The Na-rich compounds stabilized at extreme pressures (P > 200 GPa) are electrides with strong interstitial electron localization. The *C*2*/c* phase of Na_3_O is found to be a zero-dimensional electride with an insulating character. The *Cmca* phase of Na_4_O is a one-dimensional metallic electride. These findings of new compounds with unusual chemistry might stimulate future experimental and theoretical investigations.

## 1. Introduction

Sodium and oxygen are among the most abundant elements in the solar system [1]. Sodium readily interacts with oxygen, typically producing Na_2_O, in which Na and O have oxidation states +1 and −2, respectively. Sodium can also react with oxygen to form sodium peroxide (Na_2_O_2_), sodium superoxide (NaO_2_), and sodium ozonide (NaO_3_), in which peroxide (O_2_^2−^), superoxide (O_2_^−^), and ozonide (O_3_^−^) groups, respectively, act as anions [2]. Na–O compounds have broad applications, e.g., as oxidizing agents, oxygen sources, and magnetic materials [3,4]. They are also the discharge products of Na–air batteries [5,6,7]. Therefore, obtaining an in-depth understanding of the structure and properties of Na–O compounds under different external conditions is of fundamental importance. The high-pressure structures and properties of these Na–O compounds have been widely investigated both experimentally and theoretically at 50 GPa [5,8,9,10,11,12].

Pressure is a powerful tool to rearrange electrons, modify chemical bonding, and create new exotic materials [13,14,15,16,17,18]. The rapid development of structure prediction has facilitated the discovery of pressure-stabilized compounds with unusual stoichiometries; examples include S–H [19,20,21], Na–Cl [22], Xe–O/Fe [23,24], and La–H [25,26]. Some of them have subsequently been successfully synthesized [27,28]. Alkali metal sodium is a typical element showing an intriguing structure and properties under compression. For instance, the observed anomalous insulativity in the first high-pressure electride Na-hP4 [29] led to a research boom on high-pressure electrides. Similarly, many novel stoichiometries and chemical properties have been found in the Na–Cl system under high pressure [22]; Na_3_Cl, Na_2_Cl, Na_3_Cl_2_, NaCl_3_, and NaCl_7_ are theoretically stable and show unusual bonding and electronic properties. Remarkably, the most inert element, He, has shown the ability to form a compound, Na_2_He, at pressure greater than 113 GPa [30]. Oxygen also shows intriguing structures and oxidation states under compression. It is also the third most abundant element in the Earth′s crust; hence, understanding its behavior under extreme pressures provides important insights into planetary interiors and oxidation chemistry [2]. A recent experimental and theoretical study found an unconventional pressure-stabilized divalent ozonide CaO_3_ crystal with intriguing bonding; its existence has profound geological implications [31]. Recent discoveries of iron oxides with unusual oxidation states (FeO_2_ [32], Fe_2_O_3_ [33], and Fe_5_O_6_ [34]) are also notable.

Considering the intriguing stoichiometries, structures, and electronic properties of Na- and O-related substances under compression, binary compounds formed by Na and O atoms show relatively simple high-pressure behaviors. Thus, one might wonder whether new phenomenon (new stoichiometries, structures, and electronic properties) can be observed for Na–O systems at elevated pressures. Thus, this work reports a systematic search for crystal structures of different Na–O stoichiometries at pressures of 50–300 GPa with the aim of finding compounds unavailable under ambient conditions. Four new stoichiometries are predicted to be thermodynamically stable: NaO_5_, NaO_4_, Na_4_O, and Na_3_O. Four new phases (*P*2*/m* and *Cmc*2_1_ NaO_2_ and *Immm* and *C*2*/m* NaO_3_) of known stoichiometries are also found. The O-rich stoichiometries show the remarkable feature of having all the O atoms existing as quasimolecular O_2_ units and being metallic. Calculations of the O–O bond lengths and Bader charges are used to explore the electronic properties and chemical bonding of the O-rich compounds. The Na-rich Na–O compounds stabilized at extreme pressures (P > 200 GPa) are electrides with strong interstitial electron localization. The *C*2*/c* phase of Na_3_O is found to be a zero-dimensional (0D) electride with an insulating character. The *Cmca* phase of Na_4_O is a one-dimensional (1D) metallic electride.

## 2. Computational Methods and Details

Structure searching for the Na–O system was performed using Crystal Structure Analysis by Particle Swarm Optimization (CALYPSO) [35,36,37], an established method that has successfully predicted high-pressure structures in many systems [38,39,40,41]. The underlying structural relaxations and electronic structure calculations were performed using the Vienna ab initio Simulation Package (VASP) [42] with the Perdew–Burke–Ernzerhof generalized gradient approximation functional [43]. We used the projector augmented wave (PAW) [44] method to describe the valence electrons of the Na (2*s*^2^2*p*^6^3*s*^1^) and O (2*s*^2^2*p*^4^) atoms. We use a kinetic energy cutoff of 400 eV and k-point sampling with 0.3 Å^−1^ grid spacing. Each structure searching calculation generated 1200–1500 structures. After the structure searching, a kinetic energy cutoff of 1000 eV and dense k-point sampling with grid spacing of 0.1 Å^−1^ were used to ensure that enthalpy calculations were well converged to ~1 meV/atom. Phonon calculations with a supercell were performed using the PHONOPY code [45]. Electron localization functions (ELFs) were drawn using VESTA software [46], and Bader’s quantum theory was adopted to calculate charge transfer [47].

## 3. Results and Discussion

### 3.1. Stable Na–O Compounds at High Pressure

Our extensive searches for crystal structures of Na_x_O_y_ (x = 1–4 and y = 1–5) considered pressures of 50, 100, 200, and 300 GPa with simulation cells having up to four formula units (f.u.) for each fixed composition using CALYPSO methodology, which allows efficiently finding stable structures given only the chemical composition [35,36,37]. All the candidate structures were relaxed using the VASP code [42], and thermodynamic stabilities were systematically investigated by calculating the formation enthalpies relative to Na and O at the corresponding pressure. The enthalpy of formation per atom is calculated as follows:

Δ*H* = [*H*(Na*_x_*O*_y_*) − *xH*(Na) − *yH*(O)]/(*x* + *y*) (1)

**Figure 1:**
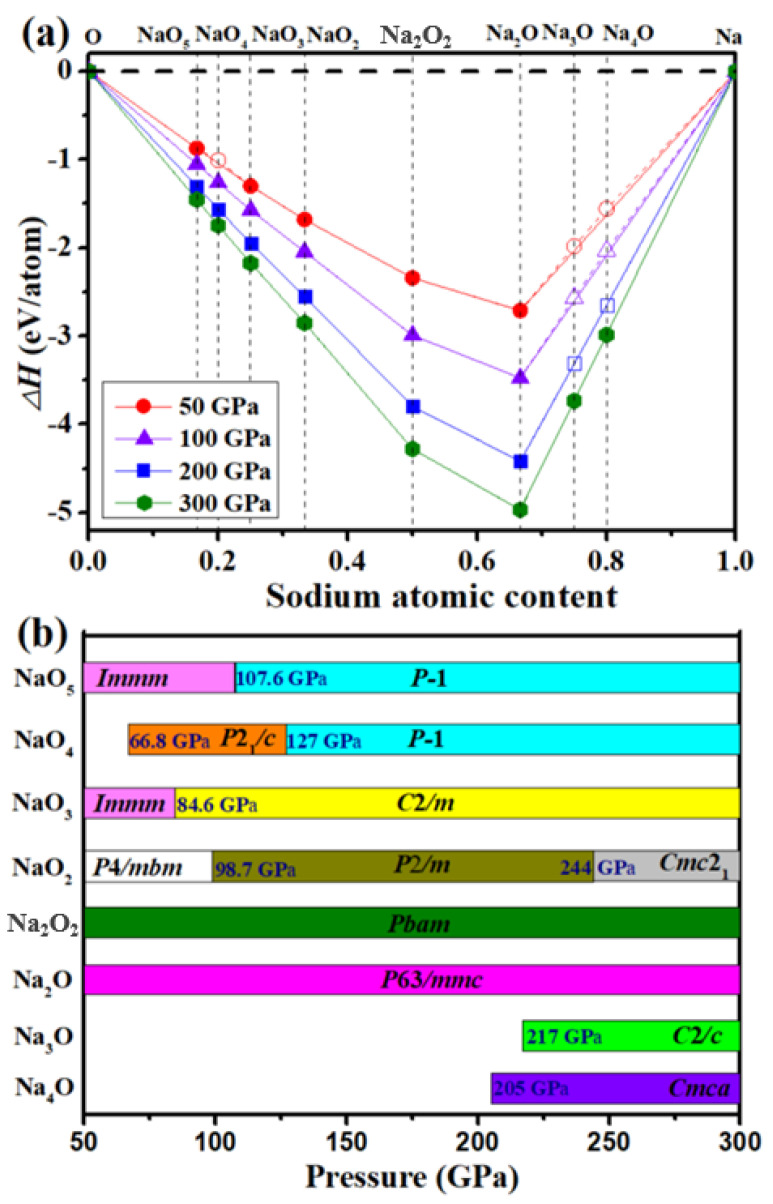
(**a**) Calculated formation enthalpy (Δ*H*) for each Na_x_O_y_ composition relative to O and Na at 0 K. Solid symbols represent stable compounds, and open symbols represent metastable compounds. (**b**) Pressure range and structure of each stable compound. Na adopted the following structures: Na−bcc, Na−fcc, Na−cI16, Na−oP8, and Na−hP4 [29,48,49,50,51], and O adopted ζ phases with *C*2*/m* symmetry [52].

Convex hull data at 0 K under different pressures (summarized in Figure 1a) show the stable compounds and phases as lying on the global stability line of the convex hull. The open symbols on the dotted lines represent unstable or metastable, and they decompose into other Na_x_O_y_ compounds or elemental solid Na and O.

The calculated formation enthalpies of Na_x_O_y_ compounds in Figure 1a show that already known compounds of Na_2_O, Na_2_O_2_, NaO_2_, and NaO_3_ lie on the convex hulls at the whole pressure range. NaO_3_ is known to possess an *Im*2*m* phase at ambient pressure [11]. Yang [5] reported NaO_2_ to have three stable high-pressure phases (*Pnnm*, *Immm*, and *P*4/*mbm*) between 0 and 50 GPa. It has been reported that Na_2_O_2_ is stable in *Amm*2 (distorted *P*$\bar{6}$_2_*m*) and *P*2_1_/*c* phases at low temperature [8]. However, the *P*$\bar{6}$_2_*m* and *Pbam* structures become the most stable at elevated temperature under pressures up to 300 GPa [9]. Na_2_O undergoes phase transition from a cubic antifluorite (*Fm*$\bar{3}$*m*) structure to an orthorhombic anticotunnite structure (*Pnma*), and then to a Ni_2_In-type (*P*6_3_/*mmc*) structure [10].

At elevated pressures, four new phases (*P*2/*m* and *Cmc*2_1_ NaO_2_, *Immm* and *C*2/*m* NaO_3_) of the compounds with known stoichiometry were found. Moreover, new stoichiometries NaO_5_, NaO_4_, Na_4_O, and Na_3_O became thermodynamically stable. An *Immm* phase of NaO_5_ stabilized at 50 GPa, and then transformed to a *P*-1 phase at 107.6 GPa. NaO_4_ stabilized in a P2_1_/c phase above 66.8 GPa, and then transformed to a *P*-1 phase at 127 GPa. Na_3_O stabilized at 217 GPa in a *C*/2*c* structure, and Na_4_O stabilized at 205 GPa in a *Cmca* structure. To provide further information potentially useful for experimental synthesis, Figure 1b shows calculated pressure–composition diagrams of the stable Na–O compounds. All of the predicted compounds were dynamically stable without any imaginary phonon modes in the whole Brillouin zone (Figure S1 in the Supplementary Materials). Table S1 gives detailed structural information, and auxiliary POSCAR files are added below Table S1 in the Supplementary Materials.

### 3.2. O-Rich Compounds

The O-rich compounds showed a remarkable feature of having all O atoms as quasimolecular O_2_ units. In addition to the previously proposed structures, new structures and stoichiometries were found, as discussed below in detail. To analyze these new structures, we calculated the O–O bond lengths and Bader charges in established structures of *C/*2*m* O_2_ [52], *Pbam* Na_2_O_2_ [9], *P*4*/mbm* NaO_2_ [5], and *P*6_3_*/mmc* Na_2_O [10] at pressures of 50–300 GPa for comparison (Table S2).

**Figure 2:**
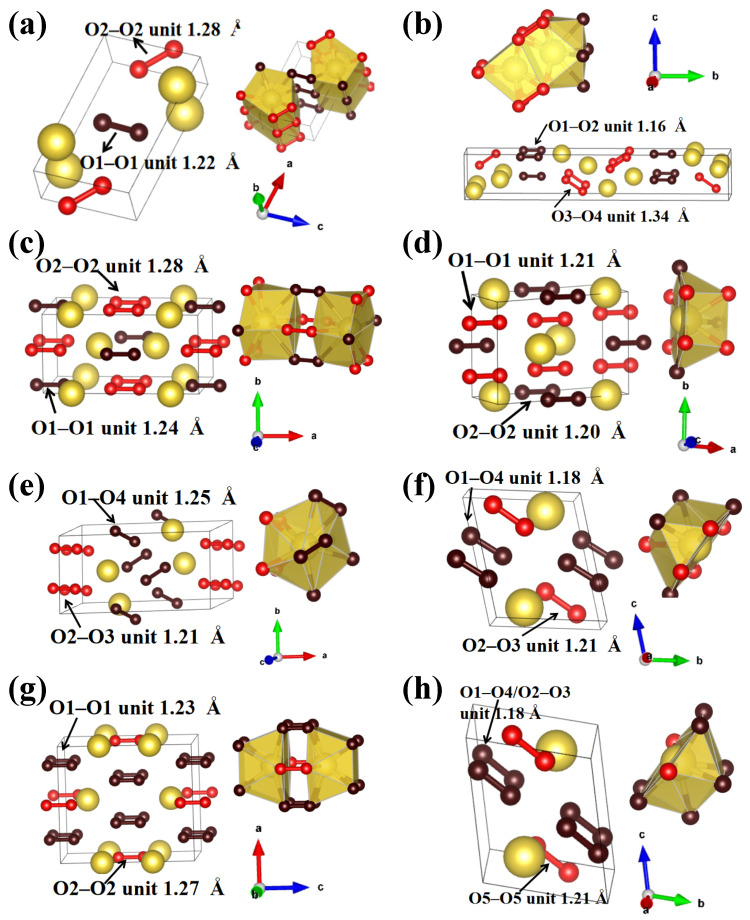
Crystal structures of the predicted O-rich Na–O compounds: (**a**) *P*2*/m* NaO_2_ at 200 GPa; (**b**) *Cmc*2_1_ NaO_2_ at 300 GPa; (**c**) *Immm* NaO_3_ at 50 GPa; (**d**) *C*2*/m* NaO_3_ at 300 GPa; (**e**) *P*2_1_/c NaO_4_ at 120 GPa; (**f**) *P-*1 NaO_4_ at 300 GPa; (**g**) *Immm* NaO_5_ at 50 GPa; (**h**) *P-*1 NaO_5_ at 300 GPa. Small spheres (dark and light red) represent O atoms; yellow spheres denote Na atoms.

For NaO_2_, in addition to three previously proposed structures (*Pnnm*, *Immn*, and *P*_4_*/mbm*) [5], two new structures were found: *P*2*/m* (2 f.u./cell) and *Cmc*2_1_ (4 f.u./cell). The former contained one Na atom at the 2 m position and two inequivalent O atoms at 2 m sites. The O–O distances were 1.22 and 1.28 Å at 200 GPa. Within this structure, each Na atom was coordinated with 10 O atoms, forming pentagonal prisms (Figure 2a). The calculated Bader charges were −0.71 for O1–O1 and −0.94 for O2–O2 quasimolecular O_2_ units, indicating that the oxidation state of the two quasimolecular O_2_ units in *P*2*/m* NaO_2_ was −1. These results confirm that this species can be viewed as a superoxide group O_2_^–^. *Cmc*2_1_ NaO_2_ stabilized above 244 GPa and consisted of two inequivalent Na atoms at the 4a position and four inequivalent O atoms at the 4a sites (Figure 2b). Within the *Cmc*2_1_ structure, Na1 and Na2 atoms were coordinated with 10 and eight O atoms, respectively. The O1–O2 distance was 1.16 Å, and the calculated Bader charges of −0.25 at 300 GPa imply an intermediate bonding situation. The O3–O4 distance was 1.34 Å, and the calculated Bader charges were −1.39 at 300 GPa, comparable to the 1.37 Å distance and −1.62 charge transfer in the peroxide (O_2_^2–^) of Na_2_O_2_ at 300 GPa (Table S2). These results confirm that the O3–O4 quasimolecular O_2_ units can be viewed as O_2_^2–^ units, which were first observed in superoxide. This indicates that the superoxide group is not maintained in NaO_2_ with increasing pressure.

An *Im*2*m* phase of NaO_3_ containing unusual ozone anions at ambient pressure was previously reported [11]. Our calculations found two new NaO_3_ phases (*Immm* (2 f.u./cell) and *C*2*/m* (2 f.u./cell)) whose O atoms existed as quasimolecular O_2_ units rather than ozone anions. The *Immm* structure contained one Na atom at the 4e position and two inequivalent O atoms at the 8 m and 4f sites (Figure 2c). The Na was coordinated with 10 O atoms; O1 was coordinated with three Na atoms, whereas O2 was coordinated with two Na atoms and one O2 atom (Figure 2c). The O2–O2 bond length of 1.28 Å is close to the distance within the superoxide group (O_2_^–^) in NaO_2_ (1.31 Å) at 50 GPa. The calculated Bader charge of the O2–O2 quasimolecular O_2_ units was −0.67, comparable to that in NaO_2_ (−0.84; Table S2). These results confirm that O2–O2 quasimolecular O_2_ units can be viewed as O_2_^–^ units with a −1 formal oxidation state. The O1–O1 distance in *Immm* NaO_3_ was 1.24 Å at 50 GPa, lying between that in neutral molecular oxygen (1.20 Å at 50 GPa) and the superoxide anion O_2_^−^ (1.31 Å at 50 GPa). The calculated Bader charge of O1–O1 quasimolecular O_2_ units was −0.39. The calculated Bader charge and O1–O1 distance suggest that the O1–O1 quasimolecular O_2_ units had an intermediate bonding situation. In the *C*2*/m* NaO_3_ structure, the Na atom was coordinated with nine O atoms, and the O–O distances were 1.20 and 1.21 Å at 300 GPa (Figure 2d). The O–O distance and Bader charges (Table S1) suggest these quasimolecular O_2_ units had an intermediate bonding situation that did not coincide with that of any known O_2_ functional group.

NaO_4_ stabilized in a *P*2_1_*/c* structure (4 f.u./cell) above 66.8 GPa. The structure had one Na atom at the 4e position and four inequivalent O atoms at the 4e sites. The O–O distances were 1.21 Å and 1.25 Å at 120 GPa. Each Na was surrounded by 11 O atoms, forming an irregular polyhedron (Figure 2e). At 127 GPa, the *P*2_1_*/c* structure transitioned to *P-*1 NaO_4_ (2 f.u./cell) with O–O distances of 1.18 and 1.21 Å at 300 GPa (Figure 2f). The O–O distances and Bader charges (Table S1) indicate that all quasimolecular O_2_ units in *P*2_1_/c and *P*-1 NaO_4_ phases had an intermediate bonding situation that did not coincide with that shown by any known O_2_ functional group. Previous studies of lithium oxides at high pressure [53,54] reported LiO_4_ phases with space groups of *Ibam* or *I*4*/mcm* at 50 GPa. The more accurate calculation used here for the Na–O system found the *P*2_1_*/c* structure to be energetically favorable at 50 GPa.

Our calculations for NaO_5_, the most O-rich Na–O composition, found two crystal structures with *Immm* (2 f.u./cell) and *P-*1 (2 f.u./cell) symmetries. The *Immm* contained one Na atom at the 4i position and two inequivalent O atoms at the 16o and 4j sites (Figure 2g). The Na atom was coordinated with 10 O atoms, and each O atom was coordinated with two Na atoms and one O atom. The O–O distances (1.23 and 1.27 Å at 50 GPa) were intermediate between those in neutral molecular oxygen (1.20 Å at 50 GPa) and in the superoxide anion O_2_^−^ (1.31 Å at 50 GPa). Compressing *Immm* NaO_5_ transformed it into *P-*1 NaO_5_ at 107.6 GPa. This structure had each Na atom coordinated with nine O atoms (Figure 2i). The O–O bond lengths (1.18 and 1.21 Å) were much shorter than those in the *Immm* structure. The calculated Bader charges and O–O distances indicate that NaO_5_ and NaO_4_ showed similar results, implying an intermediate bonding situation of all the oxygen pairs, which did not coincide with that shown by any known O_2_ functional group. Enhancing the O content in transition metal oxides can generally obtain O_2_^2−^ or O^2−^ groups [55,56,57], whereas the high-oxygen-content Na–O compounds of the present study did not give these groups.

**Figure 3:**
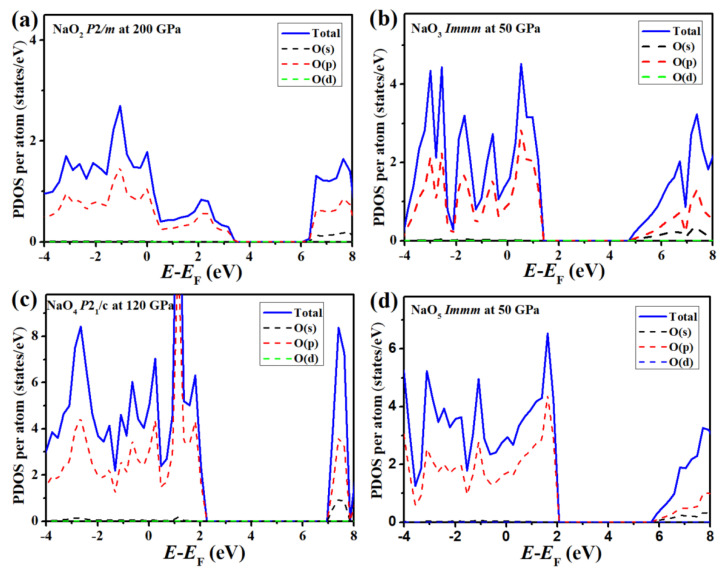
PDOS of the predicted O−rich Na−O compounds: (**a**) *P*2*/m* NaO_2_ at 200 GPa; (**b**) *Immm* NaO_3_ at 50 GPa; (**c**) *P*2_1_*/c* NaO_4_ at 120 GPa; (**d**) *Immm* NaO_5_ at 50 GPa. The PDOS of Na is not shown, as it had negligible contributions near the Fermi energy. The Fermi energy (E_F_) was set to zero.

To understand the electronic structures of O-rich compounds, we calculated the projected density of states (PDOS). All the O-rich compounds shared similar features. Thus, we present the PDOS of the lower-pressure structures for each compound in Figure 3 as representatives and provide the PDOS of higher-pressure structures in Figure S2 in the Supplementary Materials. As can be seen form Figure 3, the O 2*p* states dominated the valance bands, while the contribution of Na to the valance states was negligible since electrons were transferred from Na to O. However, there were insufficient Na atoms to donate their electrons to fully occupy the O 2*p* states. All these O-rich compounds were electron-deficient, and the partially occupied O 2*p* electronic bands led to metallicity. All the metallic O-rich compounds were nonmagnetic, similar to YO_3_ [55], LiO_2_ [54], and NaO_2_ [5]. This can be attributed to pressure-induced magnetic collapse [58].

### 3.3. Na-Rich Compounds

**Figure 4:**
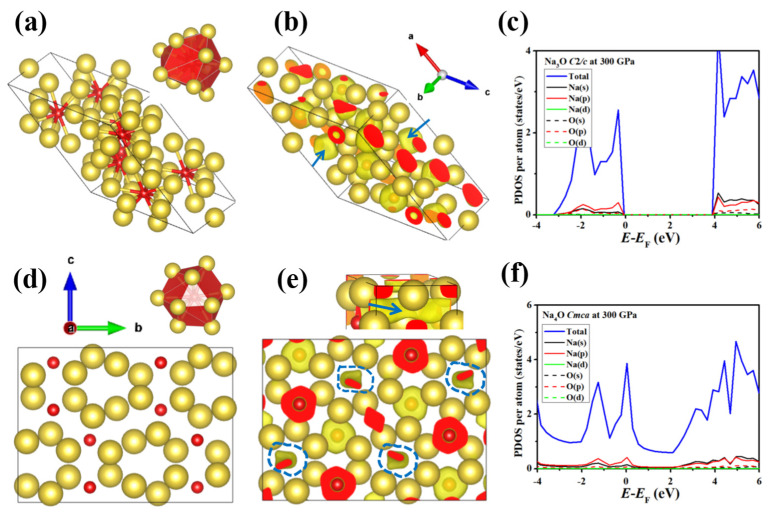
(**a**,**d**) Crystal structures, (**b**,**e**) ELF, and (**c**,**f**) PDOS of predicted Na-rich Na–O compounds at 300 GPa: (**a**–**c**) *C*2*/c* Na_3_O and (**d**–**f**) *Cmca* Na_4_O. O atoms are represented by bright red spheres; yellow spheres denote Na atoms. The interstitial electron regions are marked with blue arrows and dashed lines.

Compression stabilized Na_3_O at 217 GPa with a *C*2*/c* structure and Na_4_O at 205 GPa with a *Cmca* structure. In the *C*2*/c* Na_3_O structure, the O atom was surrounded by 11 Na atoms, forming a 17-faced polyhedron. The distance between neighboring oxygen atoms was 2.65 Å at 300 GPa (Figure 4a). In *Cmca* Na_4_O, the coordination number of O increased to 12, and neighboring oxygen atoms were 2.73 Å apart at 300 GPa (Figure 4d). Both phases had all O atoms as oxide ions rather than quasimolecular O_2_ units. The Na-rich Na–O materials are naturally electron-rich systems, making them potential candidate electride materials. Electrides have some electrons localized at interstitial regions, rather than being attached to atoms, and these electrons behave as anions [59]. According to the dimensionality of the anionic electrons and corresponding interstitial spaces where the electrons are trapped, electrides can be classified into zero-dimensional (0D), one-dimensional (1D), two-dimensional (2D), and three-dimensional (3D) electrides [60]. Miao and Hoffmann attributed the formation of high-pressure electrides to external pressure inducing changes in energy between the interstitial space and the valence orbitals of atoms [15,61].

The calculated Bader charges for the *C*2*/c* Na_3_O and *Cmca* NaO_4_ phases show that charge was transferred from Na to both O and interstitial spaces (Table S1). The electrons provided by Na atoms were first captured by O atoms to reach a stable eight-electron closed-shell configuration. Further electrons from the Na were then trapped in the interstitial spaces, favoring electride formation. Subsequent ELF analysis characterized the localization of the excess electrons. The ELF maps for *C*2*/c* Na_3_O and *Cmca* Na_4_O with an isosurface value of 0.7 at 300 GPa (Figure 4b,e) clearly show electrons localized in the interstices of the crystal, suggesting electride formation. Anionic electrons in the *C*2*/c* Na_3_O electride were limited to 0D (Figure 4b). The anionic electrons in 0D electrides are completely localized in the void of the crystal and do not contribute to the conductivity of the system. Thus, 0D electrides tend to form semiconductors or insulators, such as the insulating phase of Na-hP4 [29] at 320 GPa and semiconductor phase of Li-aba2-40 [39] at 70 GP. The other structure, *Cmca* Na_4_O, was a 1D electride in which anionic electrons were delocalized in a channel, in which the electrons could move along the channel, leading to a metallic nature. The electronic properties of both structures were explored through PDOS calculations (Figure 4c,f). *C*2*/c* Na_3_O was clearly insulating due to Na–O ionic bonding and the localized 0D interstitial electrons. However, *Cmca* Na_4_O was metallic; the states around the Fermi level were all mainly contributed by the Na 3*p* and 3*s* orbitals and O 2*p* orbitals (Figure 4f). Similar electride suboxides, Li_6_O [54] and Mg_3_O_2_ [62], have also been reported at high pressure. Na_4_O is a non-superconducting metal electride.

To show the general trend of electronic properties of the Na–O compounds, we summarize the DOSs at the Fermi level for various Na–O compounds at 300 GPa as a function of Na content in Figure S3 in the Supplementary Materials. It can be seen that all the O-rich compounds were metallic due to their electron-deficient character as aforementioned. For the Na_2_O_2_ and Na_2_O compounds, the octet rule was achieved as the peroxide O_2_^2−^ group or O^2−^ anions acquired exactly two electrons from the two Na atoms, leading to an insulating character. The Na-rich compounds, Na_3_O and Na_4_O, should be metallic due to the existence of excess electrons. However, the formation of the 0D electride made Na_3_O an insulator.

## 4. Conclusions

In summary, we used systematic structure exploration and first-principles calculations to construct a high-pressure stability field and convex hull diagram of the Na–O system with different stoichiometries at pressures of 50–300 GPa. Four previously unknown stoichiometries (NaO_5_, NaO_4_, Na_4_O, and Na_3_O) and four new phases of known stoichiometries (*P*2*/m* and *Cmc*2_1_ NaO_2_ and *Immm* and *C*2*/m* NaO_3_) were predicted to be thermodynamically stable. Remarkably, the O-rich stoichiometries showed all O atoms to exist in quasimolecular O_2_ units in a metallic state. Calculated O–O bond lengths and Bader charges were used to explore the electronic properties and chemical bonding of the O-rich compounds. The Na-rich compounds stabilized at extreme pressures (P > 200 GPa) as electrides with strong interstitial electron localization. Electrons in *C*2*/c* Na_3_O localized to 0D, making the compound an insulator. In contrast, *Cmca* Na_4_O was revealed as a 1D electride with metallic features. This work provides guidance for further experimental studies of the properties of the Na–O system.

## Data Availability

The data presented in this study are available on request from the corresponding authors.

## Supplementary Materials

The following are available online at https://www.mdpi.com/article/10.3390/ma14247650/s1: Figure S1. Phonon dispersion curves of the predicted Na–O compounds at the respective stable pressures; Figure S2. The PDOS of the predicted O-rich Na–O compounds; Figure S3. The DOS at the Fermi level versus sodium content of Na–O compounds at 300 GPa; Table S1. Structure details of the conventional unit cell of Na_x_O_y_ from the CALYPSO structure searches at different pressures and auxiliary POSCAR files; Table S2. Calculated O–O bond lengths and Bader charges.
