# Supplementary material for: Unconventional Stoichiometries of Na–O Compounds at High Pressures
Source: Materials (Basel). 2021 Dec 12;14(24):7650. doi: 10.3390/ma14247650 (PMC8707189; doi:10.3390/ma14247650)
Supplement: Supplementary file 1 [file materials-14-07650-s001.zip › materials-1432500-supplementary.pdf]

materials

MDPI

*Supplementary Materials*

# Unconventional Stoichiometries of Na–O Compounds at High Pressures

**Lihua Yang [a,b], Yukai Zhang [a], Yanli Chen [a], Xin Zhong [a], Dandan Wang [a], Jihui Lang [a], Xin Qu [a,*] and Jinghai Yang [a,*]**

[a] Key Laboratory of Functional Materials Physics and Chemistry of the Ministry of Education, National Demonstration Center for Experimental Physics Education, College of Physics, Jilin Normal University, Siping 136000, China
[b] State Key Laboratory of Integrated Optoelectronics, College of Materials Science and Engineering, Jilin University, Changchun, Jilin, China

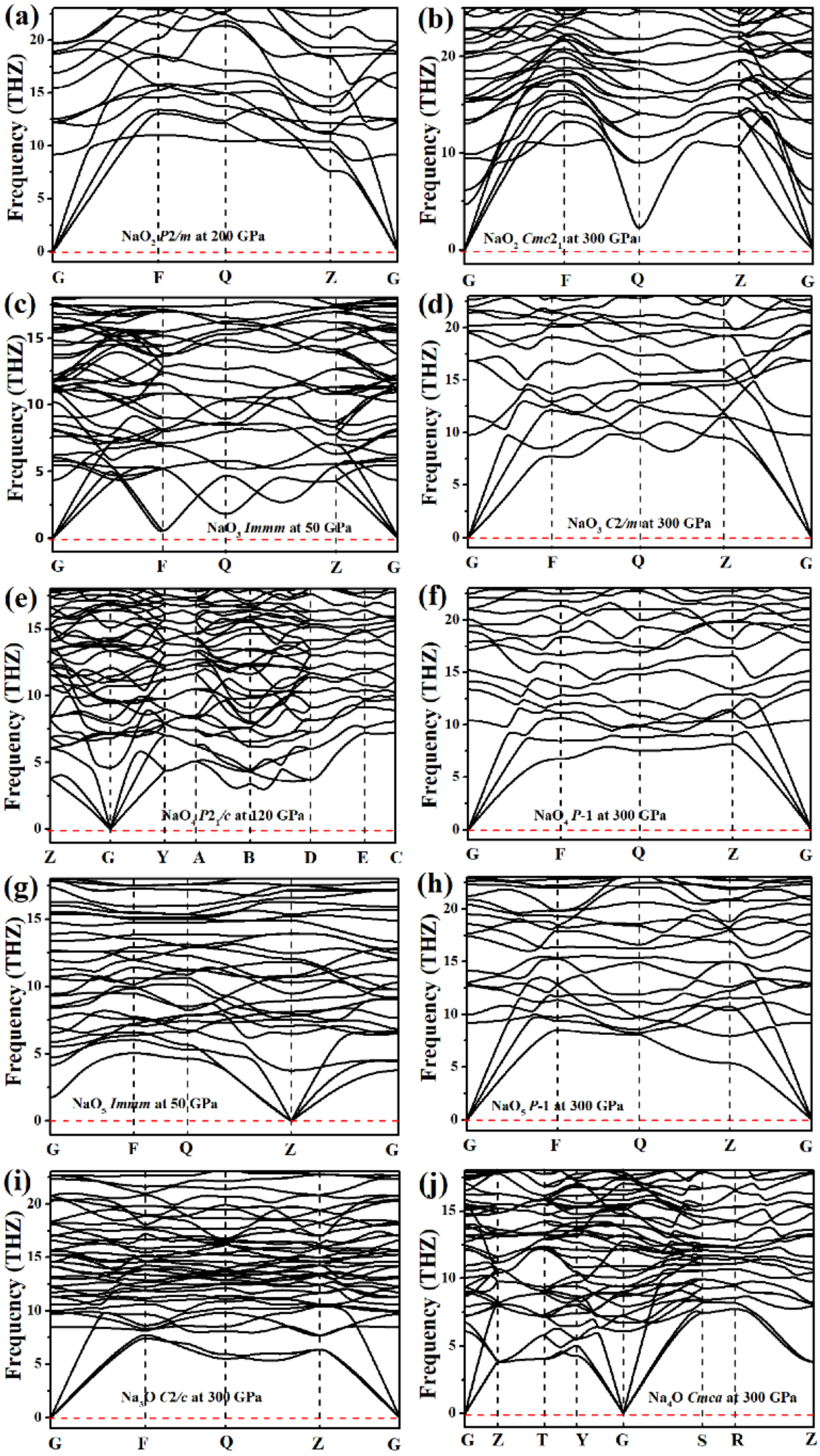


**Figure S1.** Phonon dispersion curves of the predicted Na–O compounds at the respective stable pressures. (**a**) *P*2/*m* $NaO_2$ at 200 GPa, (**b**) *Cmc*$2_1$ $NaO_2$ at 300 GPa, (**c**) *Immm* $NaO_3$ at 50 GPa, (**d**) *C*2/*m* $NaO_3$ at 300 GPa, (**e**) *P*$2_1$/*c* $NaO_4$ at 120 GPa, (**f**) *P*–1 $NaO_4$ at 300 GPa, (**g**) *Immm* $NaO_5$ at 50 GPa, (**h**) *P*–1 $NaO_5$ at 300 GPa, (**i**) *C*2/*c* $Na_3O$ at 300 GPa, (**j**) *Cmca* $Na_4O$ at 300 GPa.

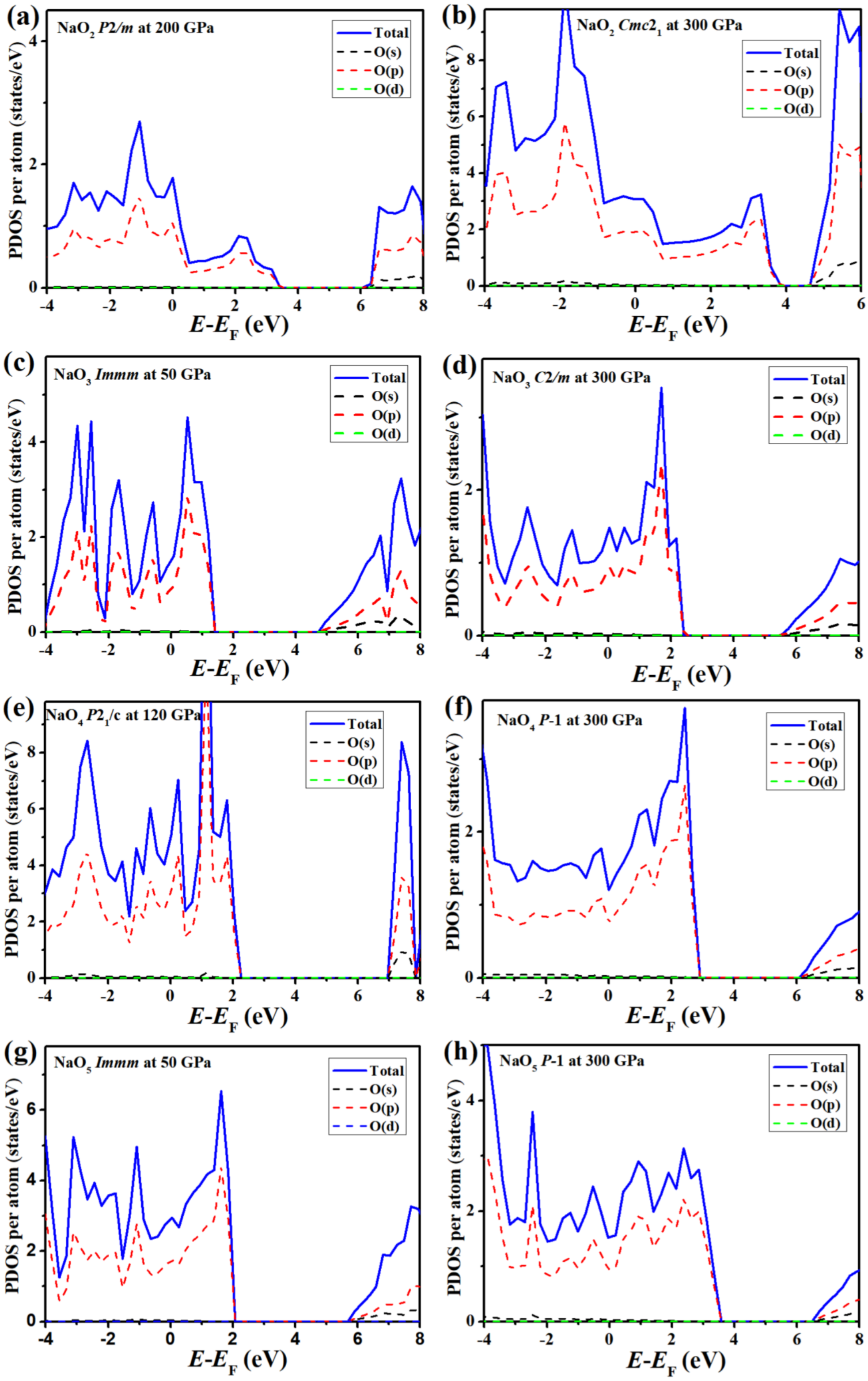


**Figure S2.** The PDOS of the predicted O-rich Na–O compounds: (**a**) *P*2/*m* $NaO_2$ at 200 GPa, (**b**) *Cmc*2$_1$ $NaO_2$ at 300 GPa, (**c**) *Immm* $NaO_3$ at 50 GPa, (**d**) *C*2/*m* $NaO_3$ at 300 GPa, (**e**) *P*2$_1$/*c* $NaO_4$ at 120 GPa, (**f**) *P*–1 $NaO_4$ at 300 GPa, (**g**) *Immm* $NaO_5$ at 50 GPa, (**h**) *P*–1 $NaO_5$ at 300 GPa,. The PDOS of Na is not shown, which has negligible contributions near the Fermi energy. The Fermi energy ( $E_F$ ) was set to zero.

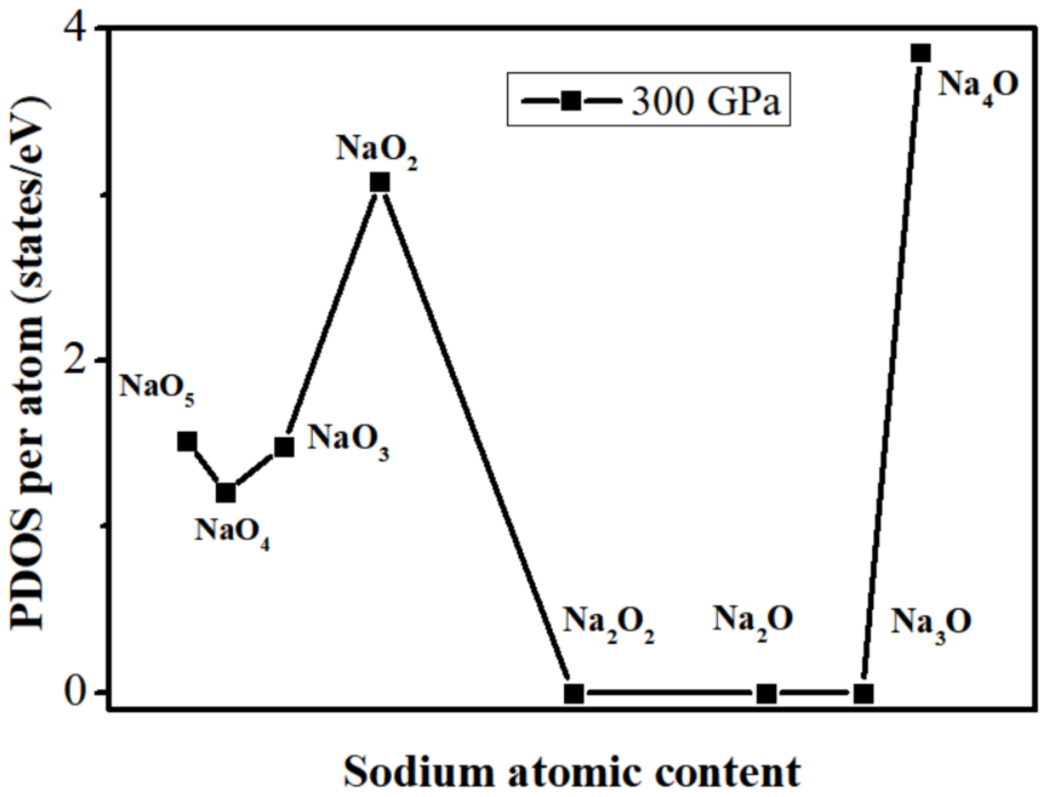


**Figure S3.** The DOS at the Fermi level versus Sodium content of Na–O compounds at 300 GPa.

**Table S1.** Structure details of the conventional unit cell of $Na_xO_y$ from the CALYPSO structure searches at different pressures.

| Pressure (GPa) | Symmetry | Z | Lattice (Å, º) | Atom | X | Y | Z | Bader Charge (\|e\|) |
|---|---|---|---|---|---|---|---|---|
| 50 | $NaO_5$ *Immm* | 4 | a=5.94 | Na(4i) | 0.00 | 0.00 | 0.69 | 0.87 |
| | | | b=4.37 | O1(16o) | 0.18 | 0.26 | -0.09 | -0.16 |
| | | | c=6.80 | O2(4j) | 0.00 | 0.50 | 0.41 | -0.23 |
| 300 | $NaO_5$ *P-1* | 2 | a=3.85 | Na(2i) | 0.62 | 0.33 | 0.14 | 0.83 |
| | | | b=4.12 | O(2i) | 0.10 | 0.94 | 0.24 | -0.16 |
| | | | c=4.16 | O(2i) | 0.75 | 0.71 | 0.43 | -0.10 |
| | | | α=103.91 | O(2i) | 0.41 | 0.07 | 0.76 | -0.18 |
| | | | β=103.79 | O(2i) | 0.25 | 0.71 | 0.42 | -0.17 |
| | | | γ=62.54 | O(2i) | 0.92 | 0.62 | 0.91 | -0.22 |
| 120 | $NaO_4$ *P2₁/C* | 4 | a=7.96 | Na(4e) | 0.72 | 0.10 | 0.76 | 0.85 |
| | | | b=4.10 | O1(4e) | 0.54 | 0.13 | 0.26 | -0.23 |
| | | | c= 4.13 | O2(4e) | 0.08 | 0.80 | 0.78 | -0.14 |
| | | | α=γ=90 | O3(4e) | 0.92 | 0.71 | 0.13 | -0.19 |
| | | | β=115.99 | O4(4e) | 0.34 | 0.02 | 0.80 | -0.29 |
| 300 | $NaO_4$ *P-1* | 2 | a=3.32 | | | | | |
| | | | b=3.71 | Na(2i) | 0.54 | 0.69 | 0.88 | 0.83 |
| | | | c=4.15 | O(2i) | 0.26 | 0.98 | 0.68 | -0.17 |
| | | | α=98.55 | O(2i) | 0.07 | 0.57 | 0.28 | -0.20 |
| | | | β=97.17 | O(2i) | 0.12 | 0.81 | 0.11 | -0.30 |
| | | | γ=108.14 | O(2i) | 0.33 | 0.23 | 0.53 | -0.16 |
| 50 | $NaO_3$ | 4 | a=6.98 | Na(4e) | 0.18 | 0.00 | 0.00 | 0.86 |

| | | | | | | | | |
|---|---|---|---|---|---|---|---|---|
| | ***Immmm*** | | b=3.54 | O1(4f) | 0.59 | 0.50 | 0.00 | -0.20 |
| | | | c= 4.84 | O2(8m) | 0.59 | 0.00 | 0.72 | -0.33 |
| 300 | **$NaO_3$** *C2/m* | 4 | a=6.36 b=3.90 c=3.31 $\alpha$=$\gamma$=90 β=111.50 | Na(4i) | 0.15 | 0.00 | 0.10 | 0.83 |
| | | | | O(8j) | 0.40 | 0.74 | 0.43 | -0.29 |
| | | | | O(4i) | 0.60 | 0.00 | 0.08 | -0.25 |
| 50 | **$NaO_2$** ***P4/mbm*** | 2 | a=b=4.40 | Na(2b) | 0.00 | 0.00 | 0.50 | 0.84 |
| | | | c= 2.40 | O(4g) | 0.61 | 0.11 | 0.00 | -0.42 |
| 200 | **$NaO_2$** *P2/m* | 2 | a=5.15 b=2.02 c=3.17 $\alpha$=$\gamma$=90 β=81.43 | Na(2m) | 0.22 | 0.00 | 0.08 | 0.826 |
| | | | | O1(2m) | 0.50 | 0.50 | 0.69 | -0.36 |
| | | | | O2(2m) | 0.91 | 0.50 | 0.39 | -0.47 |
| 300 | **$NaO_2$** ***$Cmc2_1$*** | 8 | a=2.26 b=17.21 c=2.99 | Na(4a) | 0.00 | 0.96 | 0.69 | 0.81 |
| | | | | Na(4a) | 0.00 | 0.86 | 0.95 | 0.82 |
| | | | | O1(4a) | 0.00 | 0.214 | 0.95 | -0.13 |
| | | | | O3(4a) | 0.00 | 0.28 | 0.95 | -0.12 |
| | | | | O2(4a) | 0.00 | 0.45 | 0.19 | -0.77 |
| | | | | O4(4a) | 0.00 | 0.38 | 0.45 | -0.61 |
| 50 | **$Na_2O_2$** ***Pbam*** | 4 | a=3.43 b=6.57 c=2.75 | Na(4g) | 0.15 | 0.86 | 0.00 | 0.84 |
| | | | | O(4h) | 0.63 | 0.91 | 0.50 | -0.84 |
| 50 | **$Na_2O$** ***$P6_3/mmc$*** | 2 | a=b=3.56 c=4.58 | Na1(2c) | 0.67 | 0.33 | 0.75 | 0.78 |
| | | | | Na2(2a) | 0.00 | 0.00 | 0.50 | 0.80 |
| | | | | O(2d) | 0.33 | 0.67 | 0.75 | -1.58 |
| 300 | **$Na_3O$** ***C2/c*** | 8 | a=8.14 b=5.23 c=7.10 $\alpha$=$\gamma$=90 β=143.67 | Na(8f) | 0.84 | 0.18 | 0.95 | 0.65 |
| | | | | Na(8f) | 0.16 | 0.99 | 0.32 | 0.75 |
| | | | | Na(4b) | 0.50 | 0.00 | 0.00 | 0.74 |
| | | | | Na(4e) | 0.00 | 0.37 | 0.75 | 0.71 |
| | | | | O(8f) | 0.84 | 0.83 | 0.92 | -1.58 |
| | | | | e(4e) | 0.00 | 0.67 | 0.75 | -1.09 |
| 300 | **$Na_4O$** ***Cmca*** | 8 | a= 3.15 b= 10.15 c=7.30 | Na(8f) | 0.00 | 0.04 | 0.98 | 0.59 |
| | | | | Na(8f) | 0.00 | 0.65 | 0.94 | 0.64 |
| | | | | Na(8f) | 0.00 | 0.43 | 0.82 | 0.66 |
| | | | | Na(8f) | 0.00 | 0.24 | 0.84 | 0.70 |
| | | | | O(8f) | 0.00 | 0.17 | 0.10 | -1.59 |
| | | | | e1(4b) | 0.00 | 0.00 | 0.50 | -0.81 |
| | | | | e2(8e) | 0.25 | 0.61 | 0.75 | -0.60 |

Auxiliary POSCAR files of $Na_xO_y$ from the CALYPSO structure searches at different pressures.

**1. POSCAR files of $NaO_5$ Immm at 50 GPa**

Immm

1.0

5.0174999237 0.0000000000 0.0000000000

-3.1116234265 3.9361281910 0.0000000000

-1.4963921433 -1.7049246829 4.4754159431

Na O

2 10

Direct

| | | |
|---|---|---|
| 0.000000000 | 0.688950002 | 0.688950002 |
| 0.000000000 | 0.311049998 | 0.311049998 |
| 0.445320010 | 0.173409998 | 0.091320001 |
| 0.082089998 | 0.354000002 | 0.908680022 |
| 0.917909980 | 0.826590002 | 0.271910012 |
| 0.554679990 | 0.645999968 | 0.728089988 |
| 0.554679990 | 0.826590002 | 0.908680022 |
| 0.917909980 | 0.645999968 | 0.091320001 |
| 0.082089998 | 0.173409998 | 0.728089988 |
| 0.445320010 | 0.354000002 | 0.271910012 |
| 0.500000000 | 0.907270014 | 0.407270014 |
| 0.500000000 | 0.092729986 | 0.592729986 |

## 2. POSCAR files of $NaO_5$ P-1 at 300 GPa

P-1

1.00000000000000

| | | |
|---|---|---|
| 3.8475450503549857 | 0.0326076547374572 | -0.1187895518815945 |
| 1.8693438089522370 | 3.6757187595172862 | -0.0743773644859076 |
| -0.8622788013809376 | -0.6016914974743651 | 4.0243970383689600 |

Na O

2 10

Direct

| | | |
|---|---|---|
| 0.6207059720769266 | 0.3295403895851265 | 0.1375415073951913 |
| 0.3792940279230734 | 0.6704596404148759 | 0.8624584926048087 |
| 0.1011757484660549 | 0.9404208840779162 | 0.2389365134876513 |
| 0.8988242215339426 | 0.0595791159220838 | 0.7610634565123462 |
| 0.7479319441017012 | 0.7134647510162111 | 0.4250815875441205 |
| 0.2520680558982988 | 0.2865352489837889 | 0.5749183824558841 |
| 0.4136394871512508 | 0.0662490631169277 | 0.7583812826453311 |
| 0.5863605128487492 | 0.9337509368830723 | 0.2416187173546689 |
| 0.2525279911310889 | 0.7138659968860779 | 0.4144984264997476 |
| 0.7474720388689136 | 0.2861340031139221 | 0.5855015735002524 |
| 0.9192437417834114 | 0.6151907551919322 | 0.9086666784709223 |
| 0.0807562582165886 | 0.3848092448080678 | 0.0913333215290777 |

## 3. POSCAR files of $NaO_4$ $P2_1/c$ at 120 GPa

$P2_1/c$

1.0

| | | |
|---|---|---|
| 7.9628000259 | 0.0000000000 | 0.0000000000 |
| 0.0000000000 | 4.0973000526 | 0.0000000000 |
| -1.8094352999 | 0.0000000000 | 3.7111901913 |

Na O
4 16
Direct

| | | |
|---|---|---|
| 0.717310029 | 0.998420047 | 0.260669956 |
| 0.282689979 | 0.001580000 | 0.739330053 |
| 0.282689967 | 0.498419989 | 0.239330049 |
| 0.717309996 | 0.501580011 | 0.760669960 |
| 0.536949959 | 0.134200002 | 0.756699990 |
| 0.463050004 | 0.865800012 | 0.243300019 |
| 0.463050001 | 0.634199988 | 0.743300023 |
| 0.536949973 | 0.365799983 | 0.256700034 |
| 0.084349996 | 0.795599989 | 0.275470005 |
| 0.915649953 | 0.204399996 | 0.724529971 |
| 0.915649990 | 0.295599931 | 0.224529983 |
| 0.084350000 | 0.704400011 | 0.775470042 |
| 0.916430001 | 0.704719993 | 0.633299997 |
| 0.083570007 | 0.295279978 | 0.366700012 |
| 0.083570011 | 0.204720022 | 0.866700016 |
| 0.916429991 | 0.795280007 | 0.133300065 |

## 4. POSCAR files of $NaO_4$ P-1 at 300 GPa

P-1
1.00000000000000

| | | |
|---|---|---|
| 3.3149449095408117 | -0.0000733411515787 | 0.0000458310137575 |
| -1.1560615464792656 | 3.5282663393618332 | -0.0000239407418478 |
| -0.5177798844829116 | -0.8188807670133480 | 4.0341310459697821 |

Na O
2 8
Direct

| | | |
|---|---|---|
| 0.5444921567472518 | 0.6847132114637078 | 0.8774966935684636 |
| 0.4555077892527437 | 0.3152867325362946 | 0.1225032974315321 |
| 0.2620854472316794 | 0.9806249554226828 | 0.6820237009676686 |
| 0.7379145887683235 | 0.0193750385773167 | 0.3179762900323341 |
| 0.0673763020881968 | 0.5659097896155845 | 0.2799586972513067 |
| 0.9326236689118044 | 0.4340901713844159 | 0.7200412937486890 |
| 0.1197434273644559 | 0.8058591654465146 | 0.1095534776280980 |
| 0.8802565416355450 | 0.1941408345534854 | 0.8904465363719032 |
| 0.3271942610103760 | 0.2274979607222791 | 0.5290451511638229 |
| 0.6728057129896219 | 0.7725020492777217 | 0.4709548698361753 |

## 5. POSCAR files of $NaO_3$ Immm at 50 GPa

*Immm*

1.0
4.6038999557 0.0000000000 0.0000000000
-3.2418297944 3.2690112247 0.0000000000
0.6934981103 -2.2072173041 3.9803450788
Na O
2 6
Direct
0.817969978 0.000000000 0.817969978
0.182030022 0.000000000 0.182030022
0.408769995 0.276059985 0.684830010
0.591229975 0.723940015 0.315169990
0.408770025 0.723940015 0.132710010
0.591230035 0.276060015 0.867290020
0.911339998 0.500000000 0.411339998
0.088660002 0.500000000 0.588660002

**6. POSCAR files of $NaO_3$ *C*2/*m* at 300 GPa**

*C*2/*m*
1.00000000000000
3.7307924463786426 -0.0250525382012148 0.0080660187521141
1.7137717602039169 3.3139743918074935 0.0080660185894272
-1.0437183690697875 -0.6304835471971029 3.0723970529967777
Na O
2 6
Direct
0.1515578942481781 0.1515578942482065 0.1037595860207858
0.8484421207518196 0.8484421207517912 0.8962404289792119
0.6578126768376649 0.1391703869708039 0.4334260031448807
0.8608296130291819 0.3421873531623589 0.5665739968551193
0.3421873531623376 0.8608296130291961 0.5665739968551193
0.1391703869708181 0.6578126768376364 0.4334260031448807
0.6009964513914667 0.6009963923914796 0.0766554903144296
0.3990036076085346 0.3990035776085179 0.9233445326855687

**7. POSCAR files of $NaO_2$ P4/mbm at 50 GPa**

P4/mbm
1.00000000000000
4.3949341863034155 0.0000000000000000 0.0000000000000000
0.0000000000000000 4.3949341863034155 0.0000000000000000
0.0000000000000000 0.0000000000000000 2.3977535373221124
Na O
2 4

Direct
0.0000000000000000 0.0000000000000000 0.5000000000000000
0.5000000000000000 0.5000000000000000 0.5000000000000000
0.6047593884089011 0.1047593884089011 0.0000000000000000
0.3952406385910976 0.8952406645910997 0.0000000000000000
0.8952406645910997 0.6047593884089011 0.0000000000000000
0.1047593884089011 0.3952406385910976 0.0000000000000000

## 8. POSCAR files of $NaO_2$ P2/m at 200 GPa

*P2/m*
1_10
1.00000000000000
5.1508946434599814 0.0000000000000000 0.0114513302814227
0.0000000000000000 2.0185071300362845 0.0000000000000000
0.4651728627339798 0.0000000000000000 3.1355246442100588
Na O
2 4
Direct
0.2215438581973430 0.0000000000000000 0.0755310818656554
0.7784561418026570 0.0000000000000000 0.9244689331343423
0.4957501766874586 0.5000000000000000 0.6932581943692284
0.5042498533125439 0.5000000000000000 0.3067418056307716
0.9076069877365782 0.5000000000000000 0.3855403302445950
0.0923930122634218 0.5000000000000000 0.6144596697554050

## 9. POSCAR files of $NaO_2$ $Cmc2_1$ at 300 GPa

$Cmc2_1$
1.00000000000000
8.6767857363858631 0.0000755970073746 0.0000000000000000
8.3834863229717023 2.2369126023863193 0.0000000000000000
0.0000000000000000 0.0000000000000000 2.9923809647820327
Na O
4 8
Direct
0.9637602884375980 0.9637602864702401 0.6918487128800024
0.0362397115624020 0.0362397135297599 0.1918487128800024
0.8644539296122105 0.8644493990479631 0.9523736312837130
0.1355460703877895 0.1355506009520369 0.4523736312837130
0.2139991569331841 0.2139990124797393 0.9503132924708382
0.7860008580668136 0.7860010025202584 0.4503132924708382
0.2813307563067795 0.2813291585907010 0.9520203159006044
0.7186692436932205 0.7186708414092990 0.4520203159006044

0.4476302541019450 0.4476294000126870 0.1916804917794224
0.5523697158980525 0.5523705999873130 0.6916804617794199
0.3843942791160870 0.3843917963951426 0.4484036106854177
0.6156057208839130 0.6156082036048574 0.9484036106854177

**10. POSCAR files of $Na_2O_2$ Pbam at 50 GPa**

pbam
1.00000000000000
3.4273928359842407 -0.0000000000000000 0.0000000000000000
0.0000000000000000 6.5711662897890655 0.0000000000000000
0.0000000000000000 0.0000000000000000 2.7458705451671515
Na O
4 4
Direct
0.1486960158926951 0.8578502006619845 0.0000000000000000
0.8513039661073105 0.1421497633380125 0.0000000000000000
0.3513040011073099 0.3578503096619899 0.0000000000000000
0.6486960338926895 0.6421497993380155 0.0000000000000000
0.6295976620660185 0.9075145485117455 0.5000000000000000
0.3704023379339815 0.0924854324882565 0.5000000000000000
0.8704023379339815 0.4075145855117450 0.5000000000000000
0.1295977320660172 0.5924854144882550 0.5000000000000000

**11. POSCAR files of $Na_2O$ $P6_3/mmc$ at 50 GPa**

$P6_3/mmc$
1.00000000000000
3.5639998912999999 0.0000000000000000 0.0000000000000000
-1.7819999456000000 3.0865144449000002 0.0000000000000000
0.0000000000000000 0.0000000000000000 4.5763998032000002
Na O
4 2
Direct
0.6666666679999977 0.3333333360000026 0.7500000000000000
0.3333332979999994 0.6666665950000024 0.2500000000000000
0.0000000000000000 0.0000000000000000 0.5000000000000000
0.0000000000000000 0.0000000000000000 0.0000000000000000
0.3333333360000026 0.6666666730000017 0.7500000000000000
0.6666666149999969 0.3333332979999994 0.2500000000000000

**12. POSCAR files of $Na_3O$ C2/c at 300 GPa**

C2/c
1.0

4.8364000320 0.0000000000 0.0000000000
2.0096463593 4.3991006786 0.0000000000
-4.8115436200 -3.0917793415 4.2062871148
Na O
12 4
Direct
0.155829906 0.521550059 0.444779992
0.478449941 0.844170094 0.055220008
0.844170094 0.478449941 0.555220008
0.521550059 0.155829906 0.944779992
0.673959970 0.648379982 0.822770000
0.351620018 0.326040030 0.677230000
0.326040030 0.351620018 0.177230000
0.648379982 0.673959970 0.322770000
0.000000000 0.000000000 0.500000000
0.000000000 0.000000000 0.000000000
0.133759975 0.866240025 0.250000000
0.866240025 0.133759975 0.750000000
0.501420021 0.168529987 0.416030049
0.831470013 0.498579979 0.083969951
0.498579979 0.831470013 0.583969951
0.168529987 0.501420021 0.916030049

**13. POSCAR files of $Na_4O$ Cmca at 300 GPa**

Cmca
1.0
5.3122000694 0.0000000000 0.0000000000
4.3809773291 3.0044146217 0.0000000000
0.0000000000 0.0000000000 7.3006000519
Na O
16 4
Direct
0.040690007 0.040690003 0.392799978
0.459310043 0.459310038 0.892799946
0.540689998 0.540689980 0.107200030
0.959309889 0.959309988 0.607200054
0.652009999 0.652009941 0.442639931
0.847989920 0.847989988 0.942639964
0.152009997 0.152009991 0.057360053
0.347990018 0.347989998 0.557360036
0.429340013 0.429339983 0.322270031
0.070659992 0.070659991 0.822270031

| | | |
|---|---|---|
| 0.929339915 | 0.929339973 | 0.177729969 |
| 0.570659972 | 0.570659996 | 0.677729969 |
| 0.235180025 | 0.235180008 | 0.335929999 |
| 0.264819967 | 0.264819981 | 0.835929966 |
| 0.735180017 | 0.735179997 | 0.164070017 |
| 0.764819959 | 0.764819971 | 0.664070034 |
| 0.165070017 | 0.165069989 | 0.597459986 |
| 0.334929943 | 0.334929931 | 0.097460035 |
| 0.665070017 | 0.665069969 | 0.902540014 |
| 0.834929992 | 0.834929960 | 0.402540014 |

**Table S2.** Calculated the O–O bond length and Bader charge transfer in established structures of *C*/2*m* $O_2$, *Pbam* $Na_2O_2$, *P*4/*mbm* $NaO_2$, and *P*$6_3$/*mmc* $Na_2O$ at pressures of 50–300 GPa.

| | **50 GPa** | **100 GPa** | **150 GPa** | **200 GPa** | **250 GPa** | **300 GPa** |
|---|---|---|---|---|---|---|
| *P*4/*mbm* $NaO_2$ O-O bond length (bader charge) | 1.31Å (-0.84) | 1.28Å (-0.82) | | | | |
| *Pbam* $Na_2O_2$ O-O bond length (bader charge) | 1.51Å (-1.68) | 1.47Å (-1.66) | 1.44Å (-1.64) | 1.41Å (-1.63) | 1.39Å (-1.63) | 1.37Å (-1.62) |
| *P*$6_3$/*mmc* $Na_2O$ O-O bond length (bader charge) | 3.08Å (-1.58) | 2.90Å (-1.57) | 2.79Å (-1.57) | 2.72Å (-1.56) | 2.66Å (-1.56) | 2.61Å (-1.56) |
| *C*/2*m* $O_2$ O-O bond length | 1.20Å | 1.19Å | 1.18Å | 1.17Å | 1.16Å | 1.15Å |
